# From Cells to Environment: Exploring the Interplay between Factors Shaping Bone Health and Disease

**DOI:** 10.3390/medicina59091546

**Published:** 2023-08-25

**Authors:** Samradhi Singh, Devojit Kumar Sarma, Vinod Verma, Ravinder Nagpal, Manoj Kumar

**Affiliations:** 1 National Institute for Research in Environmental Health, Bhopal 462030, India; sammradhisingh@gmail.com (S.S.); dkbiotek@gmail.com (D.K.S.); 2 Stem Cell Research Centre, Department of Hematology, Sanjay Gandhi Post-Graduate Institute of Medical Sciences, Lucknow 226014, India; 3 Department of Nutrition and Integrative Physiology, Florida State University, Tallahassee, FL 32302, USA; rnagpal@fsu.edu

**Keywords:** bone health, bone disorders, bone remodeling, gut microbiome, aging, osteoporosis, gut-bone axis, probiotics, regenerative therapy

## Abstract

The skeletal system is an extraordinary structure that serves multiple purposes within the body, including providing support, facilitating movement, and safeguarding vital organs. Moreover, it acts as a reservoir for essential minerals crucial for overall bodily function. The intricate interplay of bone cells plays a critical role in maintaining bone homeostasis, ensuring a delicate balance. However, various factors, both intrinsic and extrinsic, can disrupt this vital physiological process. These factors encompass genetics, aging, dietary and lifestyle choices, the gut microbiome, environmental toxins, and more. They can interfere with bone health through several mechanisms, such as hormonal imbalances, disruptions in bone turnover, direct toxicity to osteoblasts, increased osteoclast activity, immune system aging, impaired inflammatory responses, and disturbances in the gut–bone axis. As a consequence, these disturbances can give rise to a range of bone disorders. The regulation of bone’s physiological functions involves an intricate network of continuous processes known as bone remodeling, which is influenced by various intrinsic and extrinsic factors within the organism. However, our understanding of the precise cellular and molecular mechanisms governing the complex interactions between environmental factors and the host elements that affect bone health is still in its nascent stages. In light of this, this comprehensive review aims to explore emerging evidence surrounding bone homeostasis, potential risk factors influencing it, and prospective therapeutic interventions for future management of bone-related disorders.

## 1. Introduction

Bone is a deliberately structured mineralized connective tissue made up of minerals, cells from various lineages, mainly osteoblasts, bone lining cells, osteocytes, and osteoclasts, and a protein matrix made primarily of type 1 collagen with metabolic and structural roles [1]. It is now widely acknowledged that, in addition to its conventional functions in locomotion, protecting internal organs, harboring of bone marrow, and maintaining mineral homeostasis, bone also regulates energy expenditure and glucose metabolism as well as influences cognition and male fertility through the osteoblast-secreted hormone osteocalcin [2]. The seemingly inanimate bone is actually quite active, with osteoclasts regularly resorbing it and osteoblasts constantly neo-forming it. Bone goes through repeated cycles of modeling and remodeling to accomplish its various functions. Bone resorption or bone formation can happen independently at different places during modeling. As a result, the bone is more able to adapt to shifting mechanical demands and adjustments in bone size and shape during growth. In contrast, remodeling is a carefully controlled process of simultaneous bone formation and resorption at a particular location that preserves skeletal integrity by rebuilding aging and injured bone. Bone-forming osteoblasts from multipotent mesenchymal stem cells (MSCs), bone-resorbing osteoclasts from the monocyte/macrophage hematopoietic lineage, and osteocytes, which are former osteoblasts trapped in the bone matrix, are three different types of bone cells that are crucial for bone modeling and remodeling.

The dynamic equilibrium that maintains this functional dualism of bone in place is influenced by both extrinsic and intrinsic variables, including genetics, environmental toxins, diet, aging, gut microbiota, mechanical stress, hormone state, and others [3]. Alterations in these factors can all lead to disruptions in the process of maintaining bone homeostasis, failure to develop an efficiently strong skeleton during growth and development, increased bone resorption leading to disruption of bone architecture and lowered bone mass, increased bone density caused by a failure in bone resorption by osteoclasts, failure to repair lost bone because of abnormalities in bone formation, and mutation in the genes involved in bone physiological processes. These imbalances can lead to bone-related disorders like osteoporosis, osteoarthritis (OA), rheumatoid arthritis (RA), osteopetrosis, bone cancers, and others. In 2019, low BMD led to 438,000 fatalities and caused 16.6 million DALYs (disability-adjusted life years), showing an increase of 111.1% and 93.8%, respectively, when compared to the numbers from 1990 [4]. On a global scale, in 2019, there were about 178 million new fractures, marking a 33.4% rise compared to 1990. Additionally, there were 455 million cases of acute or persistent fracture-related symptoms, showing a 70.1% increase since 1990. Moreover, 25.8 million years lived with disability (YLDs) were attributed to fractures, representing a 65.3% rise from 1990 [4]. Furthermore, osteoporosis affects more than 200 million individuals across the globe [5]. The heightened occurrence and gravity of bone-related conditions on a global scale result from intricate interactions among factors like aging demographics, evolving lifestyles, and socioeconomic consequences. Understanding the profound consequences these ailments pose for individuals and society highlights the critical need for holistic approaches aimed at prevention, timely intervention, and proficient management. This review will examine the role of various extrinsic and intrinsic factors responsible for disturbance in bone homeostasis and leading to the development of bone disorders in addition to potential therapeutic interventions to treat these ailments in future.

## 2. Bone Homeostasis

**Figure 1:**
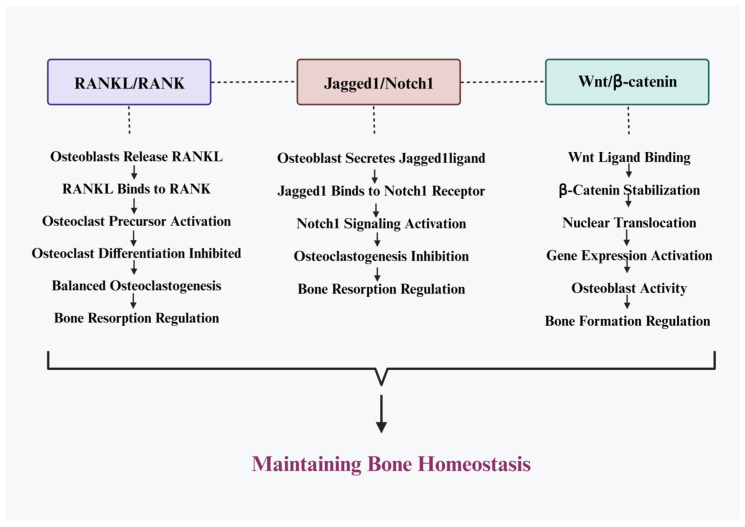
This diagram illustrates the vital roles of RANKL/RANK, Jagged1/Notch1, and Wnt/β-catenin pathways in preserving bone health, maintaining bone homeostasis, and regulating bone mineral density (BMD). Although each pathway operates autonomously, they intricately interact to collectively oversee the multifaceted process of bone remodeling. By interconnecting, these pathways ensure the perpetual renewal and durability of bone tissue.

Bone homeostasis is the process by which bone is maintained in a healthy and balanced state. The adult human skeleton has a complex process for maintaining bone homeostasis. The skeletal tissue in humans is constantly undergoing a self-regeneration process called remodeling which not only promotes calcium and phosphorus homeostasis and bone health but also enables the skeleton to respond to the body’s changing mechanical demands. In the process of bone remodeling, osteoclasts remove the distinct zones of bone and osteoblasts replace them with new bone, repairing any micro-injuries to the bone and modifying the bone niche to control mechanical strengths. The coordinated actions of bone cells, which collectively create the transient anatomical structure known as the basic multicellular unit (BMU), cause this self-renewal process to occur [6]. To maintain the health and integrity of the skeleton throughout life, the differentiation and activity of osteoblast and osteoclast must be strictly controlled. Osteoblast cells actively participate in protein synthesis and matrix secretion in order to preserve and develop new healthy bones [7]. Fully differentiated and mature osteoblasts are converted into the most common cell type, osteocytes, and placed in the bone matrix once the bone matrix has mineralized [7]. Due to their widespread distribution throughout the bone matrix, osteocytes are in a prime position to detect localized bone injury as well as variations in mechanical loading; as a result, they serve as mechano-sensors and coordinators of this bone remodeling process. Additionally, they have the ability to control bone remodeling in response to local or systemic stimuli. They influence bone remodeling by direct cell–cell interaction and the production of soluble mediators that regulate osteoclast and osteoblast recruitment, differentiation, and activity. Osteoblasts secrete osteoprotegerin (OPG), receptor activator of NF-kB ligand (RANKL), and macrophage colony-stimulating factor, to regulate the differentiation of monocyte to osteoclast. Three crucial routes that osteoblasts modulate are RANKL/RANK, Jagged1/Notch1 and Wnt/b-catenin. These signaling pathways influence BMD by regulating osteoblast and osteoclast activity (Figure 1). The maturation of osteoclast precursor occurs as a result of RANKL’s binding to its receptor RANK in the RANKL/RANK/OPG signaling pathway. A competitive antagonist to RANK, OPG inhibits osteoclastogenesis by binding to RANKL and preventing excessive bone resorption [8]. As a result, OPG and RANKL relative concentrations play a crucial role in defining bone density and strength. Numerous local (growth factors, chemokines, cytokines, etc.) and systemic factors, including hormones (such as growth, sex, and parathyroid hormones), prostaglandins, glucocorticoids, calcitonin, calcitriol, and bone morphogenetic proteins (BMP) work together to maintain bone homeostasis and regulate this remodeling process. Calcium is regulated by parathyroid hormone (PTH), calcitonin, and vitamin D. PTH and calcitonin stimulate and inhibit the release of calcium from bone, respectively, and vitamin D enhances calcium absorption from the diet. Additionally, hormones such as estrogen and testosterone can influence bone remodelling by stimulating the production of osteoblasts. In addition to hormones, other factors can contribute to the regulation of bone homeostasis. These include dietary calcium intake, physical activity, and genetic factors. Dietary calcium intake is essential for maintaining adequate calcium levels in the body, while physical activity can help to stimulate bone remodelling and increase bone density. Genetic factors can also influence bone health, such as mutations in genes coding for proteins involved in bone metabolism. When the homeostatic equilibrium shifts in favor of excessive osteoclast activity, bone degenerative disorders such as osteoporosis, Paget’s disease, and osteoarthritis (OA) develop. Nevertheless, osteopetrotic phenomena may result from poor osteoclast differentiation and/or function. Yet, increased osteoblastic activity results in an osteosclerotic phenotype, while decreased osteoblastic activity causes osteomalacia or rickets. In order to comprehend disease mechanisms and create innovative treatments for bone illnesses, it is crucial to understand the mechanism of bone homeostasis [9]. 

## 3. Intrinsic and Extrinsic Variables Affecting Bone Health

**Figure 2:**
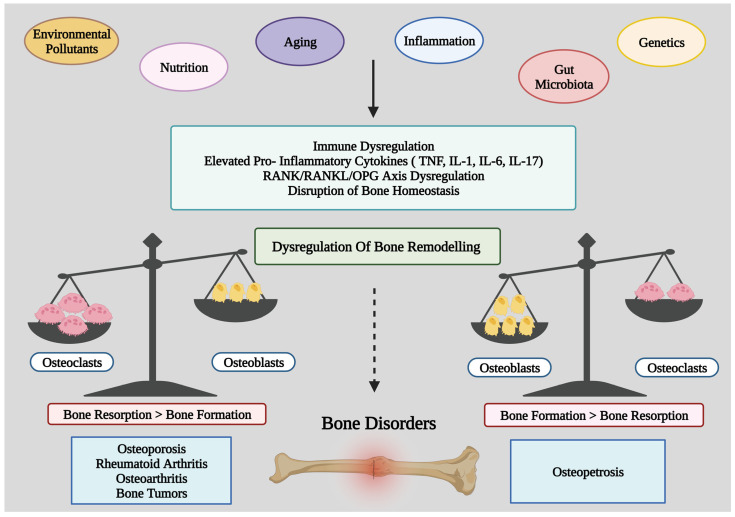
Diagram shows the putative mechanism by which intrinsic and extrinsic variables impact bone health. TNF: Tumor Necrosis Factor; IL: Interleukin; RANKL: receptor activator of NF-kB ligand; OPG: Osteoprotegerin.

Numerous factors like aging, environmental pollutants, nutrition, gut or oral dysbiosis, and genetics play an important role in maintaining bone health. These variables may contribute to the development of major bone-related disorders via dysregulating immune pathways, elevating the synthesis of pro-inflammatory cytokines, dysregulating bone homeostasis, or disrupting the bone remodeling process (Figure 2). Both the growth of bone to its maximal mass at maturity and its subsequent loss are influenced by the combination of various genetic, hormonal, environmental, and dietary factors. Controlling these variables can mean the difference between a weak and a robust skeleton. This is because even relatively small changes in bone mass can significantly affect the condition of all bones. It has been calculated that a 10% increase in bone mass might cut the risk of fractures by as much as 50% [10].

### 3.1. Aging

Maintaining bone health is of paramount importance for overall well-being, particularly as individuals age, as aging leads to a heightened susceptibility to fractures and other adverse health outcomes. Therefore, it is essential to comprehend the impact of aging on bone health and homeostasis, enabling the development of strategies aimed at mitigating the risks associated with age-related bone deterioration. The influence of age extends to both the mechanical properties and composition of bone. Irrespective of age, bones possess a hierarchical arrangement that includes diverse bone types, such as long, short, flat, and tubular, at the macroscopic level. On the tissue level, bones are organized into cortical (compact) and trabecular (woven and lamellar) structures, and on the microscopic level, they comprise cells, matrix, and mineral constituents. Further, examining the nanometer scale reveals individual crystals and collagen fibrils that compose bone’s intricate architecture [11]. The physiological functions of the bone deteriorate with age, bone weakens and loses its capacity to perform its mechanical duties, and calcium stores are frequently depleted. With aging, the delicate balance between the activities of osteoclast and osteoblast shifts in a negative direction, leading to more bone resorption and less bone formation, giving rise to bone-related disorders like osteoporosis, OA, RA, etc. Aging promotes a decrease in bone mass, which raises the risk of fractures when paired with other extrinsic and intrinsic variables. Age-related bone loss is caused by two contemporaneous but antagonistic processes at the bone surface level known as subperiosteal apposition, which takes place on the exterior of the bone, and endosteal bone resorption, which happens on the inside of the bone [12]. In both sexes, bone resorption increases linearly after the fourth decade of age due to a decrease in the formation of periosteal bone and an augmentation in the number of remodeling units within the endosteal bone [12]. Overall, these age-related changes result in trabeculae thinning, increased cortical porosity, cortical thinning, and loss of trabecular connection, all of which reduce bone quality and, consequently, bone strength [12]. Trabeculae are the lattice-like, spongy structures within bones that contribute to their overall strength and integrity. These structures provide support and help distribute forces across the bone, making them crucial for maintaining bone strength. When trabeculae thin, it means that the lattice structure becomes less dense and the individual struts or beams within the bone become narrower. Thinning trabeculae can lead to a loss of this supportive framework, further diminishing the bone’s ability to withstand external forces. Consequently, this leads to diminished rigidity and reduced tolerance for external loads [13,14]. As a consequence, the rapid decline in microarchitectural quality in both trabecular and cortical bone significantly compromises mechanical strength, accounting for approximately 90% and 75% of strength reduction during the aging process, respectively [13]. Osteoporosis, for instance, underscores this process by causing a decline in bone mass and the structural degradation of bone tissue, leading to heightened vulnerability to fractures. The deterioration of trabecular bone in osteoporosis results in localized stress accumulation and altered load-bearing patterns [13]. When trabeculae become thinner or disconnected, the bone’s capacity to uniformly distribute forces is compromised, resulting in areas of intensified stress concentration within the bone. Therefore, it is imperative to limit bone porosity, when feasible, to only the cavities necessary for biological functions such as vascular supply, marrow storage, blood cell production, biochemical signaling, transduction, and remodeling processes [13]. The process of bone loss, which is closely tied to putative age-related mechanisms, is the ultimate result of all the periosteal bone produced as we age minus all the bone permanently removed from the endosteal surface [15]. Immuno-senescence, which is the decline in the immune system associated with aging, is characterized by the production of high levels of oxidants and advanced glycation end products (AGEs). The accumulation of AGEs in bones leads to the formation of covalent cross-links with collagen and other proteins, ultimately affecting the mechanical properties of the tissue [16]. This process disturbs bone remodeling and deteriorates tissue quality by increasing stiffness and fragility. An increase in the concentration of AGEs in cortical and trabecular bone is negatively associated with bone density and mineralization. Furthermore, it impairs bone cell function and affects cortical and trabecular biomechanical properties. Senescence of B cells and T cells, osteoprogenitors, osteoblasts, and osteocytes within the bone microenvironment with aging results in increased production of key SASP factors like proinflammatory cytokines, chemokines, and extracellular matrix-degrading proteins that may contribute to the development of bone disorders [17,18].

Additionally, aging disrupts the delicate balance between collagen and mineral content in bones. Collagen is the main structural protein found in bones, providing the framework upon which minerals are deposited. As we age, there is a gradual decline in the production and quality of collagen, the protein framework that gives bones flexibility and tensile strength. Additionally, the regulation of minerals like calcium and phosphorus becomes less efficient, leading to decreased mineral density and increased porosity. This imbalance results in bones becoming more brittle and less resilient, as the diminished collagen reduces the bone’s ability to withstand bending and twisting forces, while lower mineral content reduces overall bone density and strength. The combined effect compromises bone quality, making them more susceptible to fractures, even from minor trauma or everyday activities [11]. Furthermore, telomere length, an indicator of cellular senescence, has been implicated as a marker of age-related disease progression. Numerous studies have indicated that telomere shortening impairs bone cell proliferation, differentiation, and normal function [19,20]. Telomere maintenance, which calls on the telomerase enzyme to compensate and extend telomeric sequences, can stop telomere attrition and increase cell lifespan. In this context, telomere shortening and/or telomerase deficiency can be considered potential biomarkers for the diagnosis of age-related bone loss. Bone-forming osteoblasts are primarily negatively impacted by age-related telomere dysfunction, but bone-resorbing osteoclasts are not [19]. This results in the decoupling of the bone remodeling process, which favors bone loss and the onset of several bone disorders. Since osteocytes are terminally developed osteoblasts, it is hypothesized that they may also be impacted, but little research has been done in this area. Despite the lack of data, it is feasible to speculate that telomere attrition has a causal role in the disturbance of bone homeostasis and the development of bone ailments.

#### Osteoporosis and Menopause

Aging brings about a multitude of changes within the body, and for women, one of the most pivotal transitions is menopause. Menopause, which typically occurs around the age of 50, marks the cessation of menstrual cycles and a decline in the production of estrogen—a hormone that plays a significant role in maintaining bone health. On average, women can lose about 10 percent of their bone mass during the initial five years after menopause [21]. If the bone mass before menopause was not optimal, any bone loss during this time could lead to osteoporosis. Recent data from the International Osteoporosis Foundation indicates that globally, 1 in 3 women aged 50 or older will encounter fractures due to osteoporosis during their lifetime [5]. Osteoporosis and menopause are closely linked due to the hormonal changes that occur during this life stage. Estrogen helps regulate the activity of osteoblasts and osteoclasts. It supports the production of osteoblasts while inhibiting the activity of osteoclasts, leading to a balance between bone formation and resorption. However, with the onset of menopause and the drop in estrogen levels, this delicate balance is disrupted. Osteoclast activity becomes relatively more dominant, resulting in increased bone resorption and a gradual loss of bone density [22]. As a result, postmenopausal women are at a higher risk of developing osteoporosis, a condition characterized by reduced bone mass and increased susceptibility to fractures. Bones become fragile and brittle, and even minor stresses or falls can lead to serious fractures. The spine, hips, and wrists are commonly affected.

### 3.2. Inflammation

Inflammation, a defining feature of disease and damage, tilts the bone remodeling mechanism in favor of resorption. Inflammatory mediators such as TNFα, IL-6, IL-1, glucocorticoids, bradykinin, histamine, and PGE and their associated peptides change the expression of RANK and RANKL to achieve this through interacting with osteoblasts, osteoclasts, and other immune cells.

In response to pro-inflammatory cytokines, osteoblasts secrete Macrophage Colony Stimulating Factor (MCS-F) and Monocyte Chemoattractant Protein 1, which are chemokines that attract osteoclast precursors [23]. Inflammatory mediators, such as TNFα, induce lymphocytes and endothelial cells to produce RANKL, whereas IL-1 and IL-6 stimulate osteoblasts to produce PGE2. Both of these processes may indirectly result in the production of osteoclasts and bone resorption. This would suggest that TNFα has a considerable impact on bone resorption independently of other systemic variables and that it might be possible to stop this process. Mast cells are thought to be closely related to the osteoclastic bone resorption cascade since they may also release IL-6 and TNFα. Similar to this, B lymphocytes and helper T cells seem to mediate immune responses that accelerate resorption. Local inflammatory mediators work in a bidirectional manner through contact with the periosteal, endosteal, and synovial cell surfaces, which may help partially explain the interaction between endosteal and periosteal bone remodeling [23]. Bradykinin is a vasoactive peptide that alters vascular permeability and is associated with inflammation and is believed to affect bone resorption via influencing prostaglandin synthesis in conjunction with the delayed response [24]. The resorption of bone may also be aided by other neuropeptides such as Vasoactive Intestinal Peptide and Substance P [24]. Inflammatory bone loss is usually systemic, but in diseases such as RA or periodontitis, it can also involve local bone. Furthermore, oxidative stress and the generation of advanced glycation end products (AGEs) have been identified as links between inflammation and bone destruction [16]. The vicious circle between inflammation and aging leads to the accumulation of AGEs in osteoporotic bone. Various factors such as oxidative stress, elevated glucose levels, aging, and diminished bone turnover collectively contribute to the generation and accumulation of AGEs within bone. These AGEs then initiate the onset of inflammaging, a persistent, low-level inflammation characteristic of aging [16]. Inflammaging significantly impacts various facets of bone biology, encompassing bone remodeling, mass, and strength, through a range of intricate cellular and molecular pathways. It can disturb the RANKL/OPG balance or create an imbalance between pro-inflammatory and anti-inflammatory networks, leading to disruptions in the process of bone homeostasis. This age-associated inflammation may contribute to a reduction in bone microarchitecture and loss of bone mass/strength by expanding myeloid-derived suppressor cells (MDSCs) and promoting the osteoclastogenic capacity and activity [25]. Despite the well-known routes by which inflammation affects bone, therapies that directly influence these pathways are limited. Research into therapeutics can be better targeted on lowering inflammatory-mediated bone resorption, improving outcomes, and accelerating the recovery process after injury by understanding the influence of local and systemic inflammation on bone remodelling and the specific mechanisms by which this occurs.

#### Interaction of Systemic Inflammatory Disorders with Bone Health

Systemic inflammatory diseases are a diverse group of medical conditions characterized by chronic inflammation affecting various organ systems throughout the body. Many of these diseases are known to significantly disrupt the delicate balance of bone homeostasis, leading to bone loss, increased fracture risk, and overall skeletal complications.

Conditions like rheumatoid arthritis (RA), systemic lupus erythematosus, and inflammatory bowel disease exhibit chronic and dysregulated immune responses that inadvertently impact bone metabolism [26]. In RA, the persistent inflammatory state triggers the release of cytokines such as TNF-α and IL-6, which promote osteoclast activation and bone resorption [27]. The resulting bone loss contributes to joint deformities and increased fracture risk in affected individuals. Similarly, systemic lupus erythematosus, characterized by autoantibody production and immune complex deposition, can lead to immune-mediated bone damage. Elevated levels of pro-inflammatory cytokines and altered balance between osteoblasts and osteoclasts disrupt the delicate equilibrium of bone remodeling [28]. Inflammatory bowel disease, encompassing Crohn’s disease and ulcerative colitis, also exhibits detrimental effects on bone health due to chronic inflammation and malabsorption of crucial nutrients like calcium and vitamin D [29]. These systemic inflammatory diseases underscore the intricate connection between the immune system and bone homeostasis, emphasizing the need for comprehensive management strategies that address both inflammatory and skeletal aspects to mitigate bone-related complications.

### 3.3. Gut Microbiome

The gut microbiota, comprising trillions of microbes colonizing the gastrointestinal tract, play a key role in controlling body homeostasis, including impacts on the extraintestinal and intestinal systems [30]. A balanced gut microbiota influences bone homeostasis through immunomodulation, nutrient absorption, metabolite synthesis, and regulating hormone production [31,32]. The delicate balance between bone formation and resorption can be disturbed by the gut microbiota by indirectly boosting or suppressing the activity of osteoblasts and osteoclasts, leading to osteoporosis, RA, OA, and other bone-related illnesses [33,34,35,36,37,38,39,40]. Intestinal bacteria can impact the metabolism of sex hormones, cortisol, and serotonin, as well as the level of growth factors and bone immunological status, which in turn can modify bone metabolism and, indirectly, bone mass. The gut microbiota disrupts the Wnt signaling pathway to some extent, particularly in promoting hematopoietic stem cell regeneration [41]. In addition, it also participates in the OPG pathway and inhibits RANKL [42], although the mechanism is not fully understood. Some commensal bacteria are thought to enhance the uptake of bone-related minerals such as calcium, magnesium, and phosphorus [31,43]. As intestinal physiology and gut microbial populations regulate bone metabolism [44], in both animal and human models, gastrectomy results in a reduction in BMD, and reduced gastric acid impacts the absorption and metabolism of calcium [45,46]. Moreover, some neuropeptides generated by the digestive system, such as gastric inhibitory polypeptide, gastrin, and serotonin, operate as pro-osteoporotic factors, while others, such as glucagon-like peptide (GLP)-1, GLP-2, and ghrelin, encourage bone formation or inhibit bone resorption [47,48].

In addition, during their fermentation process, intestinal microbes produce numerous bioactive compounds that are important for bone health, including B vitamins and vitamin K [31,49]. SCFAs are extra advantageous metabolites produced by the gut microbiota that have anti-inflammatory properties, preventing NF-κB activation and reducing autoimmune inflammation. SCFAs tend to regulate the gut–joint/bone axis, thereby influencing the development of bone disorders. Studies show that SCFAs, specifically propionate and butyrate, prevent osteoclastogenesis via controlling osteoclast differentiation by downregulating TRAF6 and NFATc1 [31]. Furthermore, butyrate, through the control of regulatory T cells (Tregs), is thought to encourage osteoblast differentiation and induce the development of mineralized nodules to assist bone growth [50]. Gut microbiota dysbiosis leads to disruption in the pro-osteoclastogenic pathway through a variety of mechanisms, including an increase in osteoclast precursor cells, which encourages osteoclast differentiation and suppression of anti-osteoclastogenic Th1, Th2, and Treg subsets, causing osteoclast-mediated bone loss [51,52]. Furthermore, sex hormones are essential for preserving bone homeostasis, and a lack of them can lead to gut dysbiosis, increased intestinal permeability, and elevated levels of osteoclastogenic cytokines such as RANKL, TNF, and IL-17 [53], which would increase bone loss and impact bone formation. Peripheral and central serotonin expressions are influenced by intestinal bacteria. Peripheral serotonin, which is largely produced in the gut via the catalytic activity of gut microbiota, acts as a hormone to suppress osteoblast proliferation, thereby lowering bone density and bone growth [51,54]. There is mounting proof that gut microbiome dysbiosis plays a significant role in osteoporosis development, most likely through its impact on immune system control (balancing of Th-17 and Treg cells), bone metabolism, and bone mineral absorption [55]. According to numerous studies, the gut microbiota interacts with osteoclastogenesis and bone health via a variety of mechanisms, including those mediated by microRNAs, insulin-like growth factor 1, and the immune system [56,57]. In contrast, a healthy gut microbiome may improve bone mass and treat bone disorders by limiting osteoclast differentiation and proliferation, initiating apoptosis, lowering bone resorption, or encouraging osteoblast maturation and proliferation [31]. Furthermore, to explain the interaction between the gut microbiome and OA, Hao et al. [58] proposed the existence of a “gut-joint axis”. The gut microbiota is thought to play a role in OA when pro-inflammatory bacterial LPS from the ‘leaky gut’ causes inflammation, endotoxemia, macrophage activation, and joint damage. Additionally, a study on the development of OA discovered strain-specific DNA fingerprints in mouse cartilage that were similar to human patterns, indicating the need for more research into these DNA signatures [59]. 

**Figure 3:**
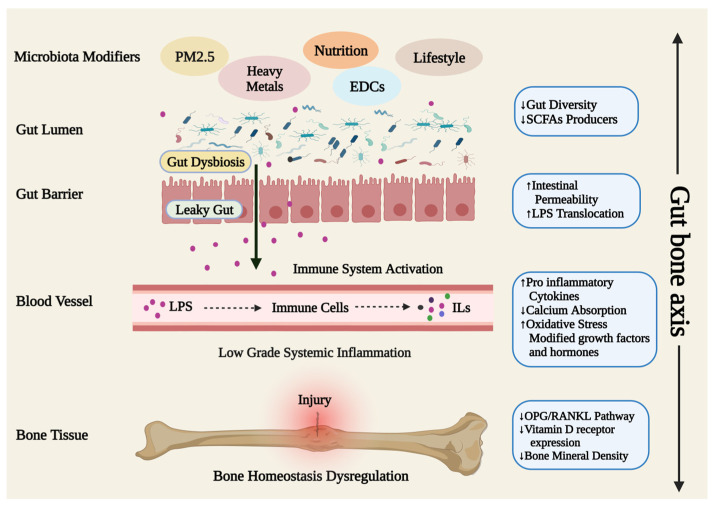
An illustration of the gut–bone axis that highlights the causes and processes starting with gut dysbiosis, leading to dysregulation in the bone homeostasis mechanism that favors osteoclasts boosting bone resorption and encouraging the emergence of bone diseases. LPS: Lipolysaccharide; ILs: Interleukins; SCFAs: Short chain fatty acids; RANKL: Receptor activator of NF-kB ligand; OPG: Osteoprotegerin; PM: Particulate Matter; EDCs Endocrine Disrupting Chemicals.

As the research continues to develop, modulating and balancing the gut microbiome using prebiotics, probiotics, synbiotics, postbiotics, or fecal microbiota transplantation is a promising component for therapeutic interventions that help mitigate or prevent bone loss [43,60,61] (Table 1). Peripheral and central serotonin (5HT) expressions are influenced by intestinal bacteria. Peripheral serotonin, which is largely produced in the gut via the catalytic activity of gut microbiota, acts as a hormone to suppress osteoblast proliferation, thereby lowering bone density and bone growth [51,54]. In [44], it was found that probiotic use can enhance intestinal flora, lower 5-HT levels, and treat bone disorders. Aberrant gut microbiota serves as biomarkers of bone metabolic activity and, thus, serves as therapeutic targets for drugs and dietary supplements to improve bone homeostasis. The gut microbiota governs the patient’s general inflammatory state and delivers TNF+ T and Th17 cells to the bone marrow, controlling bone repair and, ultimately, bone health in what is now referred to as the “gut–bone” axis [57] (Figure 3). This axis may provide new targets and therapeutic approaches for the treatment of bone cellular imbalances that might trigger immune system activation and, in turn, modify osteoclastogenesis and bone repair [57]. This claim is supported by research showing that supplementation with *Bacillus subtilis*, *Lactobacillus*, and multispecies probiotic treatment has beneficial effects not only on the human gut microbiota but also on indicators of bone metabolism [57,62,63,64]. By increasing the generation of SCFAs and promoting the overgrowth of *Lactobacillus* and *Bifidobacterium* species, *Akkermansia* and *Faecalibacterium*, probiotics enhance bone health [61]. Prebiotics have a direct anti-adherent effect on pathogens by engaging with bacterial receptors and preventing pathogen colonization. They may significantly contribute to intestinal absorption of Ca and Mg, reducing caecal pH, and increasing BMD [61]. The identification of biomarkers linked to bone disorder-related dysbiosis, as well as particular pathogens and related signal transduction pathways implicated in the disease’s etiology, will provide opportunities for the development of treatments for bone ailments that specifically target the gut microbiota. To fully comprehend the emerging role of gut microbiota in management of bone health, more research is nonetheless required.

### 3.4. Oral Microbiome

The oral microbial community serves significant functions in the development of the jawbone after birth, the natural loss of bone in the dental socket (alveolar bone) as part of physiological processes, and notably, the pathogenic loss of alveolar bone linked to oral conditions like periodontitis, apical periodontitis, and peri-implantitis. An imbalance in the oral microbiota leads to an escalation in harmful microorganisms or an increase in their disease-causing potential. 

The oral microbiota induces a catabolic impact, influencing the interplay between osteoclasts and osteoblasts that governs the remodeling of alveolar bone. This process contributes to the development of pathological bone loss [82]. An important outcome of oral microbiota dysbiosis is its correlation with alveolar bone loss, a pivotal issue in dental and periodontal well-being. This pathological alveolar bone loss, characterized by the progressive deterioration of the bone encircling the teeth, results from heightened osteoclastogenesis-driven bone breakdown and diminished osteoblastogenesis-driven bone generation. This dynamic process is jointly governed by osteoclasts and osteoblasts [82]. Based on clinical observations, animal research, and laboratory experiments, it has been found that microbial virulence factors and harmful byproducts might disrupt the body’s immune defenses against bacterial infections. This disruption can trigger a process of alveolar bone resorption [83,84]. Osteoimmunity acts as the connecting link between the microbiota and the bone [82]. In instances of dysbiosis, harmful bacteria within the dental biofilm generate virulence factors that trigger inflammation in the periodontal tissues [85]. These factors encompass LPS, which activate the host’s immune reaction, inducing the release of pro-inflammatory cytokines such as IL-1, IL-6, and TNF-α. These cytokines not only heighten inflammation within the periodontal region but also influence osteoclast function, promoting the breakdown of bone tissue [85]. The persistent and prolonged state of inflammation resulting from dysbiosis disturbs the equilibrium between formation and breakdown, ultimately resulting in a gradual loss of alveolar bone over an extended period.

#### Osteoporosis and Periodontal Disease (PD)

Osteoporosis has the potential to impact any bone within the body, including the jaws, leading to an increase in cortical thinning as individuals age. The connection between osteoporosis and oral health is not solely limited to jawbone thinning and reduced BMD. There are multiple interconnections between osteoporosis and oral conditions that have been documented, particularly concerning the relationships between osteoporosis and PD, as well as osteoporosis and disturbances in the oral microbiota [86]. PD shares numerous risk factors with osteoporosis, including factors like age, smoking and/or alcohol usage, BMI, and menopause [87]. Notably, women with osteoporosis tend to exhibit more severe cases of PD compared to women without osteoporosis. The coexistence of these two conditions significantly impacts the quality of life for affected individuals [88]. 

In a recent study by Xu et al. [89], an analysis was conducted to explore the links between osteoporosis and the risk of PD. Through a meta-analysis, the aim was to determine whether osteoporosis serves as a local indicator of bone loss or is somehow influenced by or associated with the factors that cause PD. The findings revealed a strong connection between osteoporosis and an elevated likelihood of developing PD. Notably, women with osteoporosis are at a higher risk of PD compared to men, and regardless of gender, individuals with osteoporosis face a doubled risk of PD [90]. Despite these findings, the precise underlying mechanisms behind this relationship remain undefined. Numerous hypotheses have emerged to explain how osteoporosis might hasten alveolar bone resorption in PD. Firstly, reduced BMD in alveolar bones could facilitate deeper bacterial infiltration into the expanded periodontal space, escalating local inflammation and accelerating alveolar resorption [91]; secondly, the shared elevation of proinflammatory cytokines with osteoclastic activity in osteoporosis and PD could contribute to accelerated bone loss [86]; thirdly, risk factors like smoking, diabetes, and hormone levels, which are linked to both OP and PD, might underline their interconnectedness. Conversely, other studies have examined the possibility that PD and the bacteria associated with it can directly and indirectly contribute to the initiation of osteoporosis [91]. Promising findings have emerged regarding the application of prebiotics and probiotics to rectify oral dysbiosis [92,93] and as supplementary therapeutic tools for periodontal treatments [86]. Prebiotics support the nourishment of beneficial bacteria, aiding in the restoration of a balanced and healthy oral microbiota [94]. For instance, Rosier et al. [95] highlighted the influence of nitrate as a prebiotic in altering the composition of the oral microbiome, notably by reducing periodontopathogen bacteria such as *Porphyromonas*, *Fusobacterium*, *Leptotrichia*, and *Prevotella*. Furthermore, the advantages of probiotic use have also been documented, from incorporating *Lactobacillus sp*. alongside non-surgical periodontal therapy [96] to enhancing periodontal conditions [97]. This approach not only reduces the reliance on antibiotics but also provides safe and effective support to conventional mechanical PD therapies. The positive outcomes encompass improved oral health and mitigated systemic repercussions [86]. 

### 3.5. Environmental Pollutants

Environmental toxins such as dioxins, bisphenols, polychlorobiphenyls, phthalates, poly- and perfluoroalkyl, parabens, particulate matter (PM), and heavy metals can impact bone health by interfering with bone homeostasis through a variety of processes [98,99,100,101,102]. These include hormonal imbalance, direct osteoblast toxicity, and enhancement of osteoclast activity, leading to different bone-related ailments. Pollutants can disrupt the normal balance of hormones, such as PTH, calcitonin, and vitamin D, which are responsible for the regulation of calcium levels in the body. Disruptions in these hormones can lead to an increase in calcium release from bones and a decrease in calcium absorption from dietary sources, resulting in an imbalance in calcium levels. Furthermore, some environmental pollutants can directly interfere with the production of osteoblasts. This could result in faster rates of bone deterioration and slower rates of bone formation, weakening bones and raising the risk of fractures. Additionally, some pollutants can enhance the activity of osteoclasts, further weakening bones.

Air pollution can harm bone tissue and change bone density and mineralization through a variety of processes. Numerous air pollutants can cause mild systemic inflammation that alters bone metabolism by specifically altering the development and function of osteoblasts and osteoclasts in response to the cytokines TNFα, IL-6, IL-17, and IL-1β [98]. Certain contaminants, especially specific gaseous and metal compounds, can lead to oxidative stress, thereby damaging the bone cells. By preventing osteoblast activity and MSC development into osteoblasts, exposure to heavy metals reduces bone formation. Metal exposure may lead to apoptosis or necrosis-induced cell death in osteoblasts and damage to the cytoskeleton, both contributing to the inhibition of bone formation [99]. In addition, they may enhance bone resorption by elevating the activity of osteoclast cells and macrophages/monocytes differentiation into osteoclasts. Heavy metals cause the collagen matrix to deteriorate, hinder mineralization, and promote calcium resorption, which results in the excretion of calcium and phosphorus [99]. Metal toxicity primarily affects osteoblasts by inhibiting differentiation, synthesis activity, and extracellular matrix mineralization. This interferes with the normal processes of bone remodeling and promotes the emergence of bone disorders [103]. Trace metal deposition in the extracellular bone matrix also promotes bioaccumulation and lengthens the half-life of metals in the body. This is crucial in particular cases where exposure levels are low but continuous over time. Long-term adverse effects can be the same or worse than short-term exposure to high concentrations of metals [103]. Several investigations have found that the PM component of air pollution promotes oxidative stress and systemic inflammation, both of which impede bone remodeling and subtly influence bone hormonal homeostasis via the PTH [100,101]. A few studies have shown a positive correlation between different air pollutants such as PM2.5, NOx, NO2, and carbon monoxide and bone disorders such as osteoporosis, OA, and RA [104,105,106]. Another group of pollutants, endocrine-disrupting chemicals (EDCs), can impair the functioning of the endocrine system and interfere with the physiological functions of the organ. Bone cells can be considered possible targets of EDCs because bone formation and regeneration are under complex hormonal control. Because many EDCs have xeno-estrogenic properties, the majority of these substances disrupt calcium metabolism, disrupt the balance of differentiation, proliferation, and functioning of bone cells, and cause hormonal imbalances. These effects may negatively impact the makeup of bone tissue and its mechanical and non-mechanical attributes [102]. Exposure to environmental contaminants such as lead (Pb), cadmium (Cd), phthalates, and PFAS might have a negative impact on BMD and bone homeostasis, raising the risk of bone disorders [107]. In addition, certain genetic components and air pollutants exert an additive effect on the risk of developing bone disease. Individuals’ risk of fracture or developing a bone disorder is based on their genome and is influenced by their exposome. Our understanding of how the genome and exposome interact could fundamentally alter how we approach the etiology of different bone related ailments. It is critical to restrict exposure to these substances, mostly by alterations in lifestyle, in order to lower the risk of bone disorders caused by environmental pollutants.

### 3.6. Nutrition

Nutrient intake is an important modifiable factor in bone health. Dietary components, including vitamins and inorganic minerals as well as macronutrients like protein and fatty acids, may have an impact on bone by altering the bone structure, bone metabolic rate, the endocrine and/or paracrine systems, homeostasis of calcium, and possibly other bone-active mineral elements [108]. One well-researched example of a diet that has also been specifically assessed for its effect on bone health is the Mediterranean diet. According to the findings, increased adherence to the Mediterranean diet was associated with a marginal but statistically significant rise in bone density throughout the body [109]. Additionally, supplements of nondigestible oligosaccharides such as inulin-type fructans can enhance bone health by improving intestinal barrier function, increasing absorption of calcium and magnesium, and encouraging the growth of many healthy bacteria, including *Bifidobacterium* and *Lactobacillus* [110]. 

Nutraceuticals’ impact on bone health should not be overlooked because nutrition plays a significant part in regulating it. It has been discovered that dietary supplements like butein, coronarin D curcumin, cardamonin, zerumbone, embelin, genistein, quercetin, and resveratrol contain nutraceuticals that can alter cell signaling pathways and prevent or slow down bone-related illnesses [111]. The majority of these dietary supplements are affordable, have no adverse effects, have anti-inflammatory benefits, and support bone health. Epigallocatechin, the active ingredient in green tea polyphenols, inhibits TNF-α expression, decreases osteoclast activity [112], blocks IL-6 production, and downregulates bone resorption [113]. When paired with Tai Chi, 500 mg of green tea polyphenols per day causes a net positive change in the ratio of bone formation to degradation [114]. Additionally, it has been demonstrated that in a dose-dependent manner, curcumin inhibits RANKL-induced NF-kB osteoclast formation in vitro [115]. Although nutraceuticals have been proven to minimize bone loss, enhance BMD, and significantly ameliorate bone disorders, their precise impact on osteoblast or osteoclast function and their efficacy as a therapeutic intervention in bone disorders need to be confirmed. 

### 3.7. Genetics

Skeletal strength, bone microarchitectural characteristics, and risk of developing bone disorder are significantly influenced by genetics. Genetic variables can account for up to 80% of the diversity in BMD, which is considered to be a highly heritable characteristic [116]. Several gene variants or single nucleotide polymorphisms (SNPs) have been identified that influence an individual’s susceptibility to bone diseases [116]. Numerous studies have demonstrated that 50–90% of bone mass and other qualitative features of bone are determined by genetic factors [117]. In addition to restricting bone formation in humans, heredity also controls bone shape, the rate of bone deterioration, and the skeletal system’s reaction to environmental cues like physical activity and nutrition. Although it appears that genetic factors play a role in the predisposition for bone illnesses, including osteoporosis and Paget’s disease of bone (PDB), this tendency may also be influenced by environmental variables that are yet poorly understood. Mutation in genes such as CLCN7, TCIRG1, and IKBKG is majorly responsible for various types of osteopetrosis. The formation, growth, and functioning of osteoclasts are regulated by the genes linked to osteopetrosis [118]. Osteoclasts can become aberrant or disappear when one or more of the genes linked to osteopetrosis is mutated. Furthermore, PDB is mostly influenced by genetic factors, and four genes have been shown to harbor mutations or polymorphisms that cause classical Paget’s disease and associated disorders. These include RANK-encoding TNFRSF11A, OPG-encoding TNFRSF11B, p97-encoding VCP, and p62-encoding SQSTM1 [119]. All of these genes are involved in the RANK-NFκB signaling system, and their mutations likely increase the risk of PDB by interfering with regular signaling, subsequently activating osteoclasts [119]. A very active area of research is the search for specific genes that regulate bone mass and influence the propensity to develop bone diseases. Discovery of these genetic components may lead to development of novel therapeutic interventions for bone disorders. Excitingly, it’s possible that in the near future we may be able to comprehend the genetic tendency for age-related bone loss, create improved screening techniques to identify people who are most at risk, and use genetic data to determine the best course of therapy. To comprehend the functional effects of these genetic differences, we will need to have a thorough grasp of bone structure, bone cell activity, and bone remodeling.

### 3.8. Exercise and Bone Health

Physical activity impacts bones similarly to the way it affects muscles, by enhancing their strength. Exercise holds significance in establishing robust bones during youth and sustaining bone health as we age. As living tissue, bones adapt over time due to the forces they encounter. For bone mass accumulation through osteogenic effects, bone tissues need to be subjected to mechanical loads that surpass those encountered in everyday activities [120]. Consistent exercise prompts bones to reinforce themselves and enhance density. This positive change relies on proper nutrition, including sufficient calcium and vitamin D intake. Furthermore, exercise enhances balance and coordination, which is particularly crucial as we age, as it reduces the risk of falls and subsequent fractures [121]. Regular physical activity is a key factor in promoting and preserving bone health. Various forms of exercise confer health benefits, but two notably effective techniques for strengthening bones are weight-bearing exercises and strength training, both of which stimulate bone remodeling and enhance bone strength [122]. Weight-bearing aerobic activities like walking, stair climbing, jogging, and Tai Chi stimulate bone strength [123]. Meanwhile, strength and resistance exercises, performed with or without loading, such as swimming or cycling, bolster specific muscle groups and bone density, but only within targeted body regions [120]. Exercises for improving bone strength are specific to certain areas. For instance, walking enhances bone strength in the legs and spine, though not in the wrist. Other strategies include multicomponent exercises combining various techniques like aerobics, strengthening, resistance, balance, and dance to maintain or increase bone mass. These comprehensive exercises are particularly suited for elderly individuals, often unable to engage in pure resistance training. However, their effectiveness necessitates a proportion of strengthening and resistance components. Physical exercise serves as a potent strategy for promoting bone formation in individuals with osteoporosis [122]. Mechanical loading stands as a foundational element in the accrual of bone mass. The concept, initially formulated by Frost [124] and recognized as the Mechanostat theory, proposes that bones possess an intrinsic biological mechanism that prompts bone formation in response to elevated mechanical strains, thus reinforcing the skeletal structure. This mechanism revolves around osteocytes, primarily bone cells, which can detect and react to mechanical loading. Within this framework, the protein sclerostin, produced by osteocytes, plays a pivotal role by regulating bone formation. Acting as a Wnt antagonist, sclerostin impedes the canonical Wnt/β-catenin signaling pathway, known for its influence on osteoprogenitor cell populations and mature osteoblast apoptosis. Mechanical loading reduces sclerostin expression in bones, leading to augmented osteoblastic bone formation and decreased bone resorption due to the inhibition of osteoclast activity. Notably, regions under high strain, particularly the periosteal bone surface, witness heightened bone formation alongside reduced bone turnover and porosity [120]. Consequently, mechanical loading can result in increased cross-sectional area and tissue density of bones. Moreover, this theory underscores the site-specific nature of mechanical loading’s impact on the skeletal system, with more pronounced responses observed in skeletal sites subjected to greater loading influences [120,125]. Additionally, exercise augments muscle strength, indirectly safeguarding bones by mitigating the risk of falls and fractures. Furthermore, physical activity contributes to collagen production and maintains the equilibrium between collagen and mineral content in bones. Incorporating a mix of weight-bearing and resistance exercises into one’s routine significantly aids in preserving bone quality and diminishing the susceptibility to bone-related concerns, particularly with advancing age. While exercise’s bone-building effects are evident in children and adolescents and bone-preserving effects are observed in adults, it forms only one facet of a comprehensive program to avert bone loss and lower fracture risks. Recognizing individual risk factors for osteoporosis, such as genetics and family history, holds paramount importance. A balanced diet rich in calcium, sufficient vitamin D intake, and a health-conscious lifestyle that limits excessive alcohol and nicotine usage are crucial components for lifelong bone health.

## 4. Pharmacological Interventions for Bone Disorders and Possible Consequences

The manipulation of bone metabolism using pharmaceutical interventions has brought about a revolutionary shift in the treatment of bone-related disorders. The objective of pharmacological treatment is to lower the risk of fractures. Medications designed to address bone conditions fall into two categories: antiresorptive (such as bisphosphonates, SERM, and denosumab) or anabolic (like teriparatide). Antiresorptive drugs primarily work to lower the pace of bone resorption, whereas anabolic drugs stimulate greater bone formation in comparison to resorption. 

### 4.1. Bisphosphonates

Bisphosphonates hold a primary position in the present pharmacological toolkit for countering bone loss orchestrated by osteoclasts, applied to conditions including osteoporosis, Paget’s disease of bone, bone-metastasizing malignancies, multiple myeloma, and malignancy-induced hypercalcemia. Beyond their approved applications, bisphosphonates are frequently prescribed for averting and addressing a range of skeletal issues, including low bone density and osteogenesis imperfecta [126]. Bisphosphonates find primary application in treating conditions marked by reduced bone density and heightened fracture susceptibility. They play a crucial role in restraining bone resorption and enhancing BMD. By binding to hydroxyapatite crystals in bone, bisphosphonates hinder the degradation of hydroxyapatite and impede osteoclast function, ultimately preventing excessive bone loss [126]. Due to their impact on osteoclast activity, there has been apprehension about prolonged bisphosphonate usage potentially causing “frozen bone.” This term refers to excessive suppression of bone remodeling, leading to challenges in mending skeletal microfractures and heightened skeletal fragility. This altered bone remodeling and increased microdamage have been linked to atypical femur fractures. Medication-Related Osteonecrosis of the Jaw (MRONJ) represents a significant adverse outcome, marked by non-healing exposed jawbone in the oral cavity. This condition is frequently triggered by invasive dental interventions [127]. Bisphosphonates’ influence on osteoclast activity can hinder the healing of the jawbone, potentially leading to the development of this condition.

### 4.2. Denosumab

Biological medications like denosumab operate by binding to RANKL, which hinders its connection to RANK receptors on the surface of cells within the osteoclastic lineage. This mechanism leads to the suppression of osteoclast recruitment, maturation, function, and survival, ultimately resulting in a substantial reduction in bone resorption and the consequential loss of bone mass [128]. Functioning as a bone antiresorptive treatment, denosumab’s impact on osteoblasts is predominantly indirect, achieved through the coupling of resorption and formation within the BMU [128,129]. Denosumab effectively curbs bone resorption and is employed for osteoporosis and cancer-related bone loss. It has shown the capacity to progressively elevate BMD and consistently reduce fracture incidences across skeletal sites as long as its administration persists. Its user-friendly administration and favorable safety profile make it a favorable choice for prolonged management of osteoporotic patients. Denosumab, not being embedded in the bone matrix, leads to significant and sudden alterations in bone remodeling upon discontinuation. During robust RANKL inhibition, immature preosteoclasts incapable of bone resorption accumulate within bone tissue, resulting in heightened osteoclastogenesis and RANKL release after denosumab cessation (rebound phenomenon) [128]. This triggers rapid bone loss, occasionally surpassing baseline levels, necessitating careful oversight during cessation. On a tissue level, discontinuation of denosumab compromises bone structure, evidenced by reduced cortical thickness, diminished trabecular bone volume, and increased unmineralized bone due to accelerated bone turnover [128].

### 4.3. SERMs

A distinct class of medications known as selective estrogen receptor modulators (SERMs) exhibits high affinity for the estrogen receptor, manifesting varying estrogen agonist and antagonist properties according to the specific target organ. Notably, certain SERMs, like raloxifene and bazedoxifene, exert estrogenic effects within bone, thereby curbing bone loss, enhancing BMD, and reducing the risk of vertebral fractures [130,131]. Particularly in the context of bone loss and osteoporosis, SERMs interact with the estrogen receptor to influence bone equilibrium. This interaction entails downmodulating osteoclast activity through a transforming growth factor-β3-dependent mechanism, thereby decreasing bone resorption [132]. This effect contributes to the prevention and treatment of osteoporosis. While the clinical usage of SERMs can yield various side effects, such as hot flashes and leg cramps for raloxifene, more uncommon yet severe effects like venous thromboembolism (including deep vein thrombosis, pulmonary embolism, and retinal vein thrombosis) have also been associated with raloxifene usage [132]. Patient adherence to SERM use is generally better compared to bisphosphonates. Overall, SERMs demonstrate an acceptable long-term safety profile; however, some side effects may become more apparent after approximately eight years of use. Given these attributes, SERMs are currently regarded as a favorable choice for managing osteoporosis in postmenopausal women.

### 4.4. Teriparatide

Teriparatide is a potent osteoanabolic agent composed of the first 34 amino acids of human parathyroid hormone (PTH), designed as a recombinant fragment [133]. Continuous exposure to PTH, as seen in conditions like hyperparathyroidism, leads to increased bone resorption compared to bone formation. On the other hand, intermittent exposure to low-dose PTH, such as through daily use of teriparatide, triggers bone formation to a greater extent than bone resorption. The anabolic effects of intermittent PTH occur through several mechanisms: (1) increased production of growth factors that promote bone formation, such as insulin-like growth factor 1 (IGF1) and fibroblast growth factor 2 (FGF2); (2) influencing the wnt/β-catenin signaling pathway that supports bone growth by reducing the levels of sclerostin, a wnt-antagonist; and (3) boosting the activity of Runx2, a key factor in the differentiation of osteoblasts. These processes lead to higher survival and quantity of osteoblasts, contributing to the growth of new trabecular and cortical bones [133]. Teriparatide has been subject to numerous clinical trials that have confirmed its effectiveness and safety as a treatment for osteoporosis. Apart from its approved applications for osteoporosis, research and reports suggest potential advantages of teriparatide in various areas, including fracture healing, hypoparathyroidism, osteonecrosis of the jaw, and periodontal disease [134]. Teriparatide is generally well-tolerated by patients. Short-term reported side effects include nausea, headache, dizziness, and orthostatic hypotension. Changes in calcium metabolism, primarily hypercalcemia and hypercalciuria, are frequent. The most serious concern related to teriparatide therapy is the potential risk of skeletal carcinogenicity, particularly osteosarcoma [133,135].

### 4.5. Medication-Related Osteonecrosis of the Jaw (MRONJ): A Prominent Ramification of Bone-Modulating Medications

Amid the transformative progress in bone-modulating drug therapies, the emergence of MRONJ underscores the need for comprehensive patient management strategies. MRONJ is a severe adverse effect predominantly associated with bisphosphonates and denosumab and, more recently, with other anti-resorptive agents [136]. The pathophysiology of MRONJ remains intricate, involving compromised bone remodeling and impaired blood supply, creating a susceptible environment for necrosis. The incidence of MRONJ, though relatively low, poses significant clinical challenges, including delayed healing, pain, and potential infection. Its management requires a multidisciplinary approach, encompassing accurate diagnosis through clinical, radiographic, and histopathological assessments. Conservative strategies include antimicrobial therapy, pain management, and meticulous oral hygiene. In cases of advanced MRONJ, surgical interventions like sequestrectomy, resection, and tissue grafting are considered to alleviate symptoms and promote healing [137]. Researchers showed that the combination of antibiotic therapy, surgery, leukocyte and platelet-rich fibrin (L-PRF), and photobiomodulation may effectively contribute to MRONJ management [138]. The delicate balance between managing the underlying bone disorder and mitigating the risk of MRONJ necessitates close collaboration between dental and medical practitioners to optimize patient outcomes.

## 5. Potential Therapeutic Interventions 

### 5.1. MicroRNA Therapy

**Figure 4:**
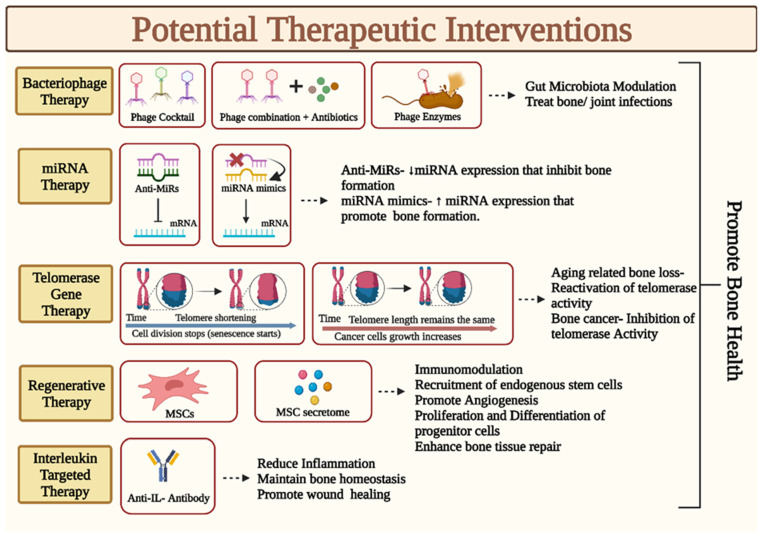
Diagram illustrates the putative mode of action of the potential therapeutic interventions that could be used for promoting bone health and treating bone disorders. IL: Interleukin; miRNA: microRNA; MSC: Mesenchymal Stem Cells.

MicroRNAs (miRNAs) are crucial for bone remodeling and regeneration [139], and miRNA-based gene treatments that involve restoring miRNA activity (miRNA mimics) or inhibiting it (anti-miRs) may be effective in treating bone-related disorders. miRNA therapy can be used to increase the expression of miRNAs that are involved in stimulating bone formation and decrease the expression of miRNAs that inhibit bone formation (Figure 4). For patients with diverse bone ailments, high-throughput miRNA analysis and sequencing can be utilized to examine multiple signaling pathways and determine the optimal treatment option. The main objective is to conduct massive replication tests to identify certain miRNAs that significantly modulate bone diseases. We may also investigate the role played by certain miRNAs in the pathophysiology of bone disorders and confirm both their in vivo and in vitro activity. The possible therapeutic alternatives based on miRNAs will open up new possibilities and offer a strong substitute for the management of bone disorders and their metabolic side effects. The key obstacles to the further growth of miRNA-based treatments in clinical application are their low in vivo stability, nonspecific biodistribution, and unfavorable side effects. The key to using miRNA technology in clinical settings is to select the appropriate chemical alterations and delivery vectors that improve the biological efficacy and potency of miRNA-based drugs. Despite these obstacles, miRNA treatment shows tremendous potential for treating a range of bone ailments; however, more study is required to fully fulfill this potential. 

### 5.2. Bacteriophage Therapy

Bacteriophage therapy is a promising treatment option for modulating gut microbiota, with the potential to revolutionize the way bone disorders are treated. Studies have shown that bacteriophages can induce bacterial and metabolic changes in the gut microbiome, as well as modulate the human immune system [140]. In bone cancer treatment, bacteriophages could have a role in killing carcinogenic bacteria and introducing toxins to the tumor microenvironment. Furthermore, phage therapy has been used successfully in both “cocktail” and “personalized” formulations for the treatment of bone and joint infections for a very long time [141]. Phage therapy is anticipated to become an important supplemental or alternative therapeutic option in the future, particularly in clinical situations where bio-film-based antibiotic tolerance emerges in the midst of the escalating antimicrobial resistance challenge. While it has some drawbacks, the advantages of this novel therapy make it an exciting area of research. With further study, bacteriophage therapy may become a safe and effective treatment for bone disorders, allowing patients to lead healthier and more active lives.

### 5.3. Interleukin-Targeted Therapy 

Interleukins (ILs) are involved in various biological processes, and the use of substances that attach to ILs and change their activity is a component of IL-targeted therapy. This can help to reduce inflammation and promote bone healing. We predict that the use of IL-10 will be a unique treatment approach in bone loss illnesses since it can help maintain bone mass by inhibiting osteoclastic bone resorption and stimulating osteoblastic bone growth [142]. It might be a particularly alluring medical alternative for treating bone loss brought on by inflammation. IL-20, IL-6, and other pro-inflammatory cytokines could increase chemotaxis, angiogenesis, and inflammation and disturb the delicate balance between osteoclastogenesis and osteoblastogenesis [143]. In the future, disorders associated with IL-mediated bone loss may be treatable by inhibiting the activity of these ILs utilizing monoclonal antibodies (mAb) or small compounds. In order to delay the progression and lessen the severity of the multifactorial bone disorders, combining anti-IL-mAb therapy with already available medications may be helpful. These treatments have the potential to reduce inflammation, promote the healing of bone fractures and other bone disorders, and provide a new avenue for treating a wide range of bone diseases. Further research is necessary to fully understand their potential.

### 5.4. Regenerative Therapy 

Regenerative therapy is a promising field of medical science that has the potential to improve the health and quality of life of those with bone disorders. It uses stem cells, other regenerative cells, and biomaterials to repair and regenerate damaged or diseased tissue and organs. MSCs are a major tool in cell therapy due to their osteogenic potential and immunomodulatory, anti-inflammatory, and anti-apoptotic properties. MSCs can repair injured tissue, exhibit autocrine and paracrine functions, and modified-MSCs can transport therapeutic chemicals or genes for the treatment of bone diseases. Additionally, MSC secretome, which is collected as conditioned media (CM), is sufficient to exert the therapeutic effects of MSCs [144]. There is potential for CM to be used as an alternative to the direct implantation of stem cells for bone repairs. However, further studies are needed to assess the efficacy of this approach and the development of better methods for bone healing and regeneration. Ultimately, regenerative therapy presents a promising possibility for treating bone disorders.

### 5.5. Telomerase Gene Therapy 

Gene therapy’s modification of telomerase maintenance must satisfy two polarizing criteria to provide distinct therapeutic effects: Anticancer applications call for the repression of several genes important for telomere maintenance, including telomerase, telomerase RNA components, and shelterin complex, whereas anti-aging/regenerative applications demand gene upregulation. Transfer of genes necessary for telomere maintenance may be a potential approach to treat age-related bone diseases and revive the ability of aging tissues to regenerate. The majority of cancer patients do, however, exhibit reactivation and overexpression of telomerase, an essential step for cancer longevity. In order to produce anticancer effects in bone, telomere-associated genes in the context of cancer gene therapy must be suppressed. Telomere-associated genes are excellent candidates for gene therapy due to their extensive applicability and multifaceted therapeutic advantages for a variety of disease targets. In conclusion, telomerase gene therapy holds promise as a potential new way to treat bone diseases.

### 5.6. Ethical Concerns and Safety Issues

As the horizon of potential therapeutic options like regenerative and gene therapy, including innovations such as telomerase gene therapy, expands to address bone disorders, it brings to the forefront a range of ethical concerns and safety considerations. While these approaches offer promising solutions for previously challenging conditions, they also demand careful ethical reflection due to their capacity to modify fundamental genetic and cellular processes. A necessity arises to address concerns related to their potential long-term impacts, unforeseen repercussions, and potential off-target effects. It is imperative to prioritize the safety and effectiveness of these therapies, necessitating meticulous preclinical and clinical investigations to evaluate potential advantages and risks. Equally significant are the rigorous safety precautions necessary to navigate the complexities of manipulating genetic material and cellular mechanisms, especially within the intricate context of bone health. Balancing the potential benefits of these cutting-edge therapies with their ethical implications and safety imperatives is a critical task as we approach a future where they could reshape the landscape of bone disorder treatments.

## 6. Conclusions and Future Prospects

The skeletal system’s multifaceted role in providing structural support, facilitating movement, and maintaining mineral balance underscores its essential significance in overall human health. The intricate orchestration of bone cells is crucial for the maintenance of bone homeostasis, which is constantly influenced by a myriad of intrinsic and extrinsic factors. Emerging evidence is unveiling the complexities of bone biology at an accelerated rate, leading to an advanced understanding of bone turnover-related diseases and potential therapeutic interventions. Imbalances in bone homeostasis have been linked to common bone metabolism disorders such as osteoporosis, OA, and others. However, it is necessary to build novel and relevant in vitro, in vivo, and ex vivo models that could further elucidate the connections between the processes of bone formation and bone resorption and aid in preventing and/or ameliorating the imbalance in bone homeostasis. Drugs like bisphosphonates and denosumab can impact bone metabolism, either by enhancing formation or restraining resorption, yet they may result in potential side effects like atypical fractures and MRONJ. To counterbalance these possible consequences, forthcoming strategies concentrate on tailored, personalized treatments. Innovations in drug targets, refined formulations, and synergistic therapies strive to heighten effectiveness while curbing undesirable effects. Vigilant surveillance through advanced imaging and biomarkers enables early identification and intervention for bone health. Additionally, educating and engaging patients empowers informed decisions, supporting bone health and mitigating risks tied to prolonged drug usage. Modulation of the gut microbiome is a promising treatment approach that can increase BMD by restoring specific commensal/beneficial intestinal microbes and microbial metabolites. Nutritional supplements, including prebiotics, probiotics, postbiotics, and synbiotics, should be further investigated as practical adjunctive therapies for bone disease, particularly targeting the most relevant biochemical and signaling pathways. Future preclinical and clinical studies are indispensable to identify and adequately support the clinical management of the gut–bone axis and bone health maintenance. The novel science of “osteomicrobiology” is facilitating significant therapeutic ramifications, opening up novel therapeutic options for a variety of inflammatory bone diseases. Pharmaceuticals often target pro-inflammatory cytokines, but more comprehensive studies are needed to discover avenues to reduce inflammation-induced bone disease. Dietary supplements, including herbs, minerals, and phytochemicals, could also be included in patient regimens to support bone health, though clinical evaluation is necessary for safe use. Potential therapies such as OPG mimics, RANKL inhibition, senescent cell targeting, and gene therapy are also anticipated to facilitate investigating treatment options for bone ailments. 

## Figures and Tables

**Table 1:** List of major clinical and in vivo studies using different gut microbiome interventions for bone-related ailments.

Gut Microbiome Intervention	Study Design	Human/Animal Model	Outcome	Reference
Red clover extract (RCE) rich in isoflavone aglycones and probiotics for 12 months	Randomized Controlled Trial (RCT)	Postmenopausal osteopenia women	Attenuated BMD loss caused by estrogen deficiency. Improved bone turnover and estrogen metabolism.	[65]
Multispecies probiotic supplement for 6 months	RCT	Postmenopausal osteopenia women	Favorable effects on bone health. Slowed down the bone turnover rate.	[66]
10^10^ CFU *Lactobacillus reuteri* (L. reuteri 6475) for 12 months	RCT	Low BMD women	Reduced bone loss.	[64]
*Lactobacillus reuteri* for 1 year	RCT	Elderly women with low BMD	Reduced bone loss in older women with low BMD. Beneficial effects on bone metabolism.	[67]
Two daily capsules *Lactobacillus rhamnosus* (LGG^®^), *Saccharomyces cerevisiae* (boulardii), and *Bifidobacterium animalis* ssp lactis for 10 weeks	N-of-1 trial design	Osteoarthritis patients	Reduction in pain scores.	[68]
Skimmed milk containing probiotic *Lactobacillus casei* Shirota for 6 months	RCT	Knee osteoarthritis patients	Significant improvement in WOMAC (Western Ontario and McMaster Universities Osteoarthritis Index) and VAS (visual analog scale) scores. Serum levels of hs-CRP were also significantly lower.	[69]
*Bifidobacterium animalis* subsp. lactis Probio-M8 [Probio-M8] for 3 months	RCT	Postmenopausal Osteoporosis Patients	Co-administering Probio-M8 improved bone metabolism, reflected by an increased vitamin D3 level and decreased PTH and procalcitonin levels in the serum.	[70]
Prebiotics/probiotics preparations combined with zoledronic acid + calcitriol regimen for 3 months	RCT	Patients with primary osteoporosis (POP)	Improved bone metabolism and intestinal floras, and suppressed cytokines release in patients with POP.	[71]
1 × 10^9^ CFU/mL of *L. acidophilus*, *B. longum*, and *L. reuteri* for 4 weeks	RCT	Mature ovariectomized osteoporotic female Sprague Dawley rats	Significant ameliorative effect on global bone mineral content and BMD.	[72]
Probiotic *L. reuteri* (1 × 10^9^ CFU/mL) with *L. reuteri*, 3 times per week for 4 weeks	RCT	Menopausal Ovariectomized Mouse Model	Suppressed bone resorption and loss associated with estrogen deficiency.	[73]
Mice received twice/week oral gavage of 3 × 10^9^ CFU/200 μL probiotics *Lactobacillus acidophilus* or vehicle from 9 weeks to 12 weeks for advanced OA stage screening	RCT	Experimental Murine (Female C57BL/6) OA Model	Mitigated OA-associated pain, cartilage disintegration, and gut microbiota dysbiosis.	[74]
*Bacillus clausii* (BC) administered orally as a suspension of 200 µL (10^9^ CFU/mL/day) in drinking water to mice for 6 weeks post-ovariectomy.	Comparative Interventional Study Design	Postmenopausal Osteoporotic mice	Reduced levels of proinflammatory cytokines (interleukin [IL]-6, IL-17, and tumor necrosis factor-α) and increased levels of anti-inflammatory cytokines (IL-10 and interferon-γ). Inhibited bone loss in postmenopausal osteoporotic mice model via skewing the balance of Treg-Th17 cells in vivo.	[75]
2 × 10^11^ colony-forming unit (CFU)/g. of *Lactobacillus plantarum* GKM3, *Lactobacillus paracasei* GKS6 once per day for 28 days	RCT	Ovariectomized (OVX) induced osteoporotic mice model	Supplementation ameliorated bone loss in ovariectomized mice by promoting osteoblast differentiation and inhibiting osteoclast formation. Both probiotic strains inhibited osteoporosis in the OVX mice model, with *L. paracasei* GKS6 outperformed *L. plantarum* GKM3. Both GKS6 and GKM3 promoted osteoblast differentiation and inhibited RANKL-induced osteoclast differentiation via the Bone Morphogenetic Proteins (BMP) and RANKL pathways, respectively.	[76]
*L. brevis* CD2 (8 × 10^5^ CFU in 1 mm^2^ lyopatch) which were placed between the gingiva and the buccal mucosa near the ligated teeth.	RCT	Experimental periodontitis mice model	Decreased bone loss and lower expression of tumor necrosis factor, interleukin-1β, -6, and -17A as compared to placebo-treated mice.	[77]
Synbiotic—5% *w*/*w* long-chain inulin and 10^9^ spores/day *Bacillus coagulans* for 35 days	RCT	RA-induced male Wistar rats	Improved the biochemical and clinical parameters of induced RA in the rat.	[78]
FMT-GM transplanted from young rats into aged rats for 12 and 24 weeks	RCT	Aged rats with senile osteoporosis	Alleviated bone loss. Up-regulated the expression of tight junction proteins of occludin, claudin, and ZO-1 improving gut microbiome composition and intestinal barrier function.	[79]
1 × 10^9^ CFU/mL/day postbiotic preparations for 4 weeks immediately after ovariectomy.	RCT	Post-menopausal Sprague-Dawley rat model	Ameliorated bone loss resulting from estrogen deficiency.	[80]
Postbiotic—(1 × 10^7^ or 1 × 10^8^ CFU per ml) cell-free culture supernatant of *Lactobacillus curvatus* Wikim 38 (LC38-CS) for 4 days	RCT	Mice model of ovariectomy-induced post-menopausal osteoporosis	LC38-CS inhibited RANKL-induced osteoclast differentiation by the downregulation of molecular mechanisms and exerted anti-osteoporotic effects.	[81]
